# Low-Grade Endometrial Stromal Sarcoma in a 22-Year-Old Maiden Female: A Rare Case Report from Syria

**DOI:** 10.1155/2021/5578686

**Published:** 2021-07-15

**Authors:** Lana Nseir, Georget Mansour, Junior Makhoul, Luna Skaf, Mohammad Ziad Dahhan, Zuheir AlShehabi

**Affiliations:** ^1^Department of Pathology, Cancer Research Centre, Tishreen University Hospital, Lattakia, Syria; ^2^Faculty of Medicine, Tishreen University, Lattakia, Syria; ^3^Department of Obstetrics and Gynaecology, Al-Tabiat Surgical Hospital, Lattakia, Syria; ^4^Department of Pathology, Cancer Research Centre, Tishreen University, Lattakia, Syria

## Abstract

Endometrial stromal sarcoma (ESS) is a rare and challenging type of endometrial tumor, constituting only about 0.2% of all uterine malignancies and occurring in women 42–58 years of age. ESS is usually misdiagnosed as leiomyoma. They both have nonspecific symptoms, which makes the diagnosis of ESS difficult to achieve. As the ESS is infrequently encountered, optimal management is quite debatable. Here, we report a rare case of a 22-year-old Syrian female who presented with abnormal uterine bleeding; the preoperative diagnosis suggested leiomyoma while histopathological and immunohistochemical studies confirmed the diagnosis of LG-ESS stage IIA. Therefore, the treatment plan was shifted from a conservative myomectomy to a total hysterectomy with both adnexa. The aim of this case report is to draw attention to this uncommon tumor at young age of patients as well as to have awareness of the necessity to suspect this diagnosis especially with the presentation of rapid enlargement of uterine leiomyoma.

## 1. Introduction

Uterine sarcoma is a rare malignancy that usually has an aggressive clinical behavior and a poor prognosis. It includes a heterogeneous group of infrequent tumors of uterine musculature, endometrial stroma, and connective tissue [1]. Endometrial stromal sarcoma (ESS) is an extremely rare neoplasm that accounts for less than 10% of uterine sarcomas and approximately 0.2% of all uterine malignancies with annual incidence of 1–2 per million women [2]. It is traditionally separated into two subtypes, low-grade ESS (LG-ESS) and high-grade ESS [3]. LG-ESS might be mistaken for leiomyoma, as it is difficult to recognize it clinically and is more often detected postoperatively and confirmed with histopathological examinations [4].

## 2. Case Presentation

**Figure 1:**
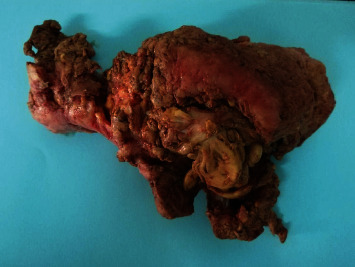
Gross examination showed an ill-defined submucosal fibroid mass excised from the uterus measuring 12 × 11 × 7 cm with few gray-pink in color polypoid masses measuring 2-3 cm that are soft to rubbery in consistency.

**Figure 2:**
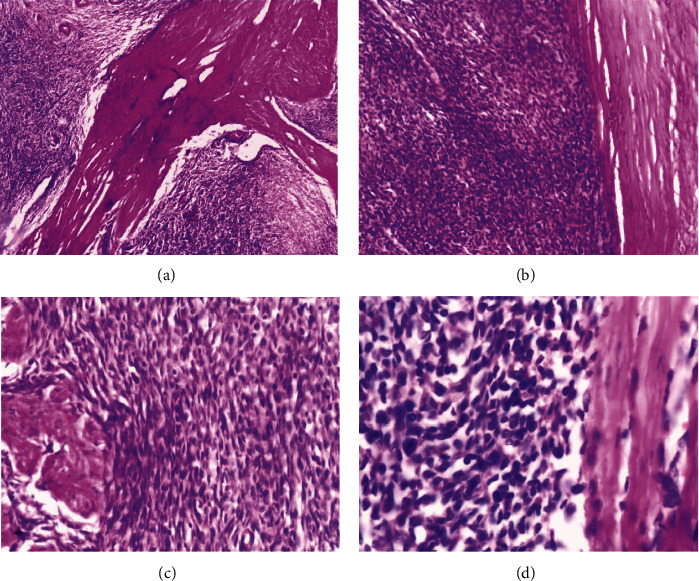
(a) Histopathology showing nodular cellular proliferation of small- to medium-sized, ovoid or spindle cells, which form interlacing bundles, whorls, and broad bands, H&E ×40. (b) Infiltration of the myometrium, H&E ×100. (c) The tumor cells exhibit only mild nuclear atypia with few mitotic figures (2-3/10 HPF), H&E ×200. (d) Higher magnification H&E ×400.

**Figure 3:**
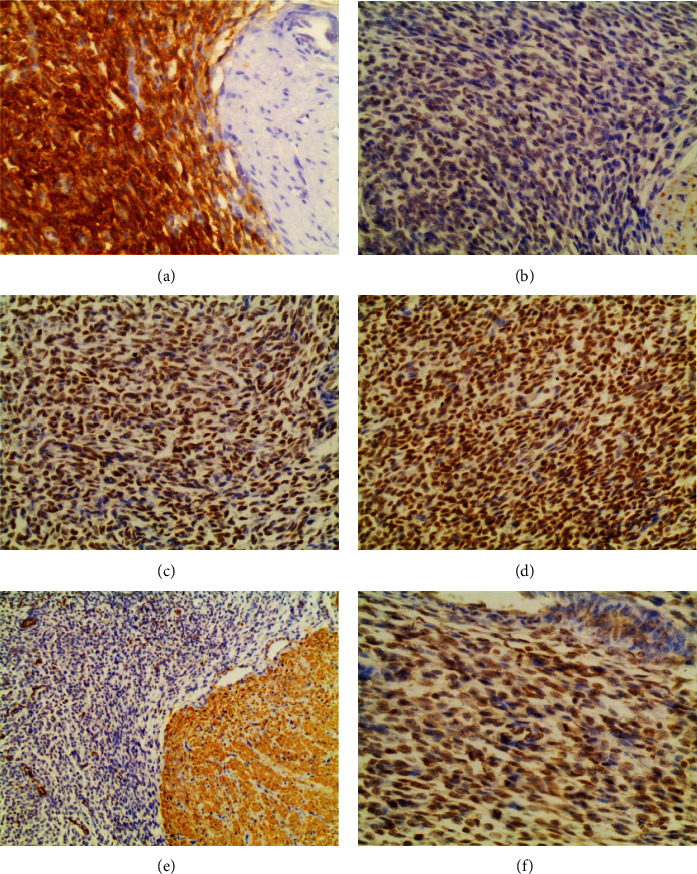
Immunohistochemistry stains showing the endometrial stromal cells: (a) diffuse and strong positivity for CD10; (b) Desmin: negative; (c) ER: positive (total score + 4/8); (d) PR: positive (total score + 6/8); (e) SMA: negative (internal control); (f) ki67: low rate ~10%.

**Figure 4:**
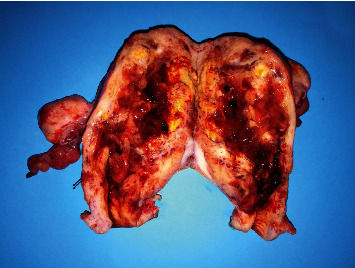
Specimen of the uterus and both adnexa measuring 12 × 8 × 6 cm. The cut sections show infiltrating ill-defined multifocal tumor nodules, arising from the endometrium, measuring ~7 × 8 cm, gray-yellowish in color, soft fleshy in consistency. The myometrium is thickened ~3-4 cm, and the ovaries contain cystic follicles.

A 22-year-old nonsmoker female presented with a five-month history of abnormal uterine bleeding. She is not sexually active and has no history of contraceptive use or any significant findings in her medical and family history. She had attained menarche at the age of 13, with regular and normal menstrual cycles. During the last 5 months, her cycles became prolonged and irregular. Pelvic ultrasound revealed an enlarged uterus with an ill-defined fibroid-like mass filling the endometrial cavity. The initial diagnosis was leiomyoma; hence, a myomectomy was performed (Figure 1). Histopathological examination revealed nodular cellular proliferation of small- to medium-sized ovoid and spindle cells, which form interlacing thick bundles and whorls. In addition, a diffuse invasion of the myometrium by the neoplastic cells was revealed (Figure 2). The morphologic features were interpreted as low-grade endometrial stromal sarcoma (LG-ESS) or cellular leiomyoma. Therefore, immunohistochemistry stains (IHC) were recommended to confirm the diagnosis. IHC showed strong positivity for CD10, ER, and PR, whereas SMA and Desmin were negative (Figure 3). This panel supported the diagnosis of low-grade endometrial stromal sarcoma (LG-ESS). A CT scan was performed in order to determine the possibility of fertility preservation treatment taking into consideration the young age of the patient. The CT scan revealed an enlarged uterus (12 × 8.5 × 7 cm), intramyometrium multinodular necrotic mass with irregular margins, and pelvic tissue reaction with mild transudate in the right iliac fossa. There was no evidence of lymph node enlargement. Based on the results of CT scan, histopathologic studies, and the patient safety, the decision was made to perform a total abdominal hysterectomy with bilateral salpingo-oophorectomy (BSO), greater omentectomy, and appendectomy (Figure 4).

Histological examination of the specimen revealed multifocal low-grade endometrial stromal sarcoma (LG-ESS) measuring (7 × 8 cm) in maximum dimension. Tumor involves serosa with direct extension to the right adnexa and internal OS of the endocervix. The greater omentum and the appendix were free of tumor cells. According to the International Federation of Gynecology and Obstetrics classification, the final diagnosis was LG-ESS (stage IIA) and the patient was referred for oncology consultation with regular and close follow-up for at least 6 months.

## 3. Discussion

Endometrial stromal sarcomas (ESSs) are a subset of uterine mesenchymal neoplasms, consisting of cells closely resembling normal proliferative endometrial stromal cells, whereas in our case, nodular cellular proliferation of small- to medium-sized ovoid and spindle cells was observed. The World Health Organization (WHO) classifies endometrial stromal neoplasms according to morphology, mitotic activity, cellularity, and the presence of necrosis into four categories [4] as follows:

- Endometrial stromal nodule (ESN)
- Low-grade endometrial stromal sarcoma (LG-ESS)
- High-grade endometrial stromal sarcoma (HG-ESS)
- Undifferentiated uterine sarcoma (UUS)

Low-grade endometrial stromal sarcoma (LG-ESS) often affects women between 42 and 58 years old. Our patient presented at 22 years, which is a rarity in itself. Although it is highly malignant, 25% of the patients are asymptomatic. Its symptoms can be vague and nonspecific and may vary from lower abdominal or pelvic pain, abnormal vaginal bleeding, to progressive menorrhagia. Moreover, it has a high tendency for recurrence and metastasis [5]. None of the imaging techniques such as ultrasound, computed tomography )CT(, and magnetic resonance imaging )MRI) can specify any characteristics of LG-ESS [6] because ESS is usually confused with leiomyoma, uterine leiomyosarcoma (LMS), or other sarcomas [3]. That is why in our case, we headed to histopathological examinations with immunohistochemistry staining [6] to confirm the diagnosis. The most frequent immunohistochemistry markers used for differentiating ESS from other tumors are CD10, Smooth Muscle Actin (SMA), Desmin, H-Caldesmon, Cytokeratin, and hormone receptors (estrogen and progesterone) [6]. For instance, stromal sarcoma cells show positivity for vimentin and ES or/and PR while they are often negative for Desmin, H-Caldesmon, SMA, and Cytokeratin [6]. In addition, diffuse and strong positivity for CD10 is a very valuable diagnostic marker of LG-ESS and could be useful in distinguishing this tumor from its histological mimics, especially cellular leiomyoma where CD10 is mostly negative [5]. Similarly, in our case, we confirmed the diagnosis of LG-ESS depending on the strong positivity of CD10, ER, and PR and on the negativity of SMA and Desmin. The first-line treatment for LG-ESS is surgical, a total hysterectomy with bilateral salpingo-oophorectomy (BSO), and excision of all grossly detectable tumors [7]. Several cases in the literature suggested some surgical maneuvers in order to preserve fertility especially for young females; however, the majority of these cases were stage I [8]. In our case, the decision of preservative treatment was extremely hard and risky because of the lack of clear guidelines in young patients and the possibility of distant metastasis. For patient safety, the decision was made to perform total abdominal hysterectomy with bilateral salpingo-oophorectomy (BSO).

## 4. Conclusion

ESS is a rare malignant tumor in this age group of women. An early diagnosis is essential because the patient survival and fertility preservation are directly related to the tumor stage. By reporting our case, we stress on the necessity of high suspicion and careful evaluation of any fibroid mass with rapid enlargement especially in young women in order to rule out this malignancy at an early stage.
